# Inhibition of Viral RNA-Dependent RNA Polymerases by Nucleoside Inhibitors: An Illustration of the Unity and Diversity of Mechanisms

**DOI:** 10.3390/ijms232012649

**Published:** 2022-10-21

**Authors:** Sailen Barik

**Affiliations:** EonBio, 3780 Pelham Drive, Mobile, AL 36619, USA; barikfamily@gmail.com

**Keywords:** RNA virus, RdRP, remdesivir, sofosbuvir, molnupiravir, transcription, COVID-19, SARS-CoV-2, hepatitis C, nucleoside analog

## Abstract

RNA-dependent RNA polymerase (RdRP) is essential for the replication and expression of RNA viral genomes. This class of viruses comprise a large number of highly pathogenic agents that infect essentially all species of plants and animals including humans. Infections often lead to epidemics and pandemics that have remained largely out of control due to the lack of specific and reliable preventive and therapeutic regimens. This unmet medical need has led to the exploration of new antiviral targets, of which RdRP is a major one, due to the fact of its obligatory need in virus growth. Recent studies have demonstrated the ability of several synthetic nucleoside analogs to serve as mimics of the corresponding natural nucleosides. These mimics cause stalling/termination of RdRP, or misincorporation, preventing virus replication or promoting large-scale lethal mutations. Several such analogs have received clinical approval and are being routinely used in therapy. In parallel, the molecular structural basis of their inhibitory interactions with RdRP is being elucidated, revealing both traditional and novel mechanisms including a delayed chain termination effect. This review offers a molecular commentary on these mechanisms along with their clinical implications based on analyses of recent results, which should facilitate the rational design of structure-based antiviral drugs.

## 1. Introduction

Important examples of highly pathogenic RNA viruses, responsible for severe diseases in humans, are shortlisted here (Table 1). In this analytical review, I have focused on the best studied ones in order to offer a molecular glimpse of their RNA-dependent RNA polymerase (RdRP), particularly relevant to interactions with substrate and inhibitor nucleotides. The *Mononegavirales* is an order of RNA viruses with negative-strand nonsegmented genomes and consists of eleven families [1], some of which are presented here (Table 1). In contrast, viruses of the *Flaviviridae* family, belonging to the Order *Amarillovirales*, contain positive-strand nonsegmented RNA genomes. Since the metazoan cells by and large lack RdRP activity, the RNA viruses encode their own RdRP, as it is essential for viral transcription and replication. In corollary, the inhibition of viral RdRP offers a lucrative target for designing specific antiviral drugs with minimal host toxicity.

Over the years, various ribonucleoside and deoxyribonucleoside analogs have been used in vitro and in vivo to inhibit transcription and replication, respectively. For example, the triphosphate derivative of cordycepin (3’-deoxyadenosine, i.e., 3’dA), i.e., 3’dATP, mimics ATP and is incorporated instead of A. However, the lack of an -OH group at the 3’ position of ribose prevents the formation of the 3’-5’ phosphodiester bond with the 5’ end of the next nucleotide, which terminates RNA synthesis [2]. Recently, it also showed strong antiviral activity against the Dengue virus [3] and a SARS-CoV-2 “variant of concern” (VOC-202012/01) [4].

In using the analogs ex vivo or in vivo (intact cells or animals), their triphosphate forms are not useful, since the triphosphates are labile and the cell membranes in general lack transport mechanisms for organic phosphates. Instead, the nucleosides without phosphate groups, and often conjugated with other moieties for desirable properties, are employed, using what is often called “ProTide” technology, detailed later (Section 3.1). The free nucleosides are enzymatically phosphorylated inside cells by kinases; when conjugated nucleosides are used, the conjugate group is removed in the plasma or inside cells, and then the free nucleoside is phosphorylated, an example of which is illustrated with remdesivir (See later). Thus, a nucleoside or its conjugate is pharmacologically referred to as “prodrug” that is inactive but undergoes “metabolic activation” to generate the active drug (i.e., the nucleotide form), which affects the cognate polymerase.

Nucleoside analogs have received particular attention as antivirals due to the fact of their ability to serve as substrates for viral RNA-dependent RNA polymerase (RdRP) by acting as nucleoside mimics [5,6,7]. Collective evidence, detailed later, shows that their mechanisms of action are broadly of two kinds: (a) Act as a chain terminator, thereby abrogating RNA synthesis immediately or soon after they are incorporated in the nascent RNA chain. An example of this category is remdesivir [8,9,10,11,12,13]. (b) Cause mutation(s): In an alternative mechanism, the analog does not cause cessation of RNA synthesis but is actually incorporated into the product RNA. The base-pairing property of the analog, however, is different from that of the nucleoside it replaced, and as a result, when the product RNA serves as a template in the subsequent cycles of RNA synthesis, the analog promotes misincorporation, resulting in multiple mutations in the viral RNA [14]. This eventually leads to the inhibition of the virus, a process sometimes referred to as “error catastrophe” or “lethal mutagenesis” [15,16,17,18]. Several NAs, notably, molnupiravir, favipiravir, and ribavirin, are examples of this class. The selected examples of both classes are presented in this review.

**Table 1:** Representative RNA viruses that are inhibited by nucleoside analogs ^1^.

Virus	Inhibitory Analog (Drug Name)
Order *Mononegavirales* (−sense RNA genome):	
Family *Paramyxoviridae*	
Parainfluenza virus (PIV)	Remdesivir [19]
Hendra virus (HeV)	Remdesivir [19]
Nipah virus (NiV)	Remdesivir [19]
Family *Filoviridae*	
Ebola virus (EBOV)	Remdesivir [8,9]; favipiravir [20]
Marburg virus (MARV)	Remdesivir [21]; favipiravir [22]
Family *Pneumoviridae*	
Respiratory syncytial virus (RSV)	Remdesivir [8,9]
Order *Amarillovirales* (+sense RNA genome):	
Family *Flaviviridae* (NS5; processed from polyprotein) [23]	
Hepatitis C virus (HCV)	Remdesivir, sofosbuvir [24]
Dengue virus (DENV)	Cordycepin [3]
Yellow fever virus (YFV)	Remdesivir [25]
Tick-borne encephalitis virus (TBEV)	Remdesivir [23]
Zika virus (ZIKV)	Sofosbuvir [24]
West Nile virus (WNV)	Sofosbuvir [26]
Order *Nidovirales* (+sense RNA genome):	
Family *Coronaviridae*	
SARS-CoV	Remdesivir [12,13]; cordycepin [4]; EIDD-1931 [6]
MERS-CoV	Remdesivir [27]
Order *Picornavirales* (+sense RNA genome):	
Family *Picornaviridae*	
EV71 (+sense RNA genome)	Remdesivir [28]

^1^ This is a highly selective list and not comprehensive of all inhibitors or all viruses that are inhibited by the listed inhibitor. Similarly, each virus family comprises scores of viruses that differ in sequence, pathology, and host tropism and, therefore, only a few examples in each family are listed here, particularly the ones that are relevant for public health. For space constraints, only selected references are cited here, but several others can be found in more comprehensive reviews [5,6,29]. As a rule, the nucleoside analogs are administered in the blood as the inactive “prodrug” forms, which are then biochemically converted into the “active” triphosphate form for incorporation into the RNA product. Most analogs go by multiple names, given by different researchers and pharmaceutical companies, a few of which are mentioned here. Often times, the prodrug, the intermediate(s) in the conversion pathway, and the final triphosphate are given different names, as illustrated later for remdesivir and molnupiravir. CoV, coronavirus; SARS, severe acute respiratory syndrome; MERS, Middle Eastern respiratory syndrome; EV71, enterovirus 71.

## 2. Major Nucleoside Analogs with an Antiviral Effect

As mentioned before, the active form of a nucleoside analog (henceforth abbreviated as NA) is the triphosphate, but it is administered as a prodrug that is inactive and activated metabolically. The steps for the activation are very similar, as illustrated later, for remdesivir, molnupiravir, and sofosbuvir. In brief, the prodrug is synthesized with substitutions that increase the lipophilicity and improve the bioavailability. When administered in the body, the substitutions are removed in multiple steps of catalytic and spontaneous reactions to produce the monophosphate form. The latter is phosphorylated further by cellular kinases to generate the final triphosphate form that is active and recognized by RdRP as a nucleotide analog. In an alternative pathway, the monophosphate is dephosphorylated to generate the nucleoside, which can be fully phosphorylated to the triphosphate. It is fair to say that the renewed awareness of the antiviral effect of the NAs owes its origin to remdesivir, an adenosine analog (See later), when it was repurposed to inhibit SARS-CoV-2 (severe acute respiratory syndrome coronavirus-2), the causative agent of the deadly pandemic COVID-19 (coronavirus disease 2019). Remdesivir, sold under the brand name Veklury, was previously developed for the treatment of hepatitis C virus (Table 1). Highly comprehensive recent reviews, listing essentially all nucleoside analogs and their clinical antiviral applications are available [5,6,7], whereas this review presents their diverse molecular mechanisms, using selected analogs as representatives of each.

**Figure 1:**
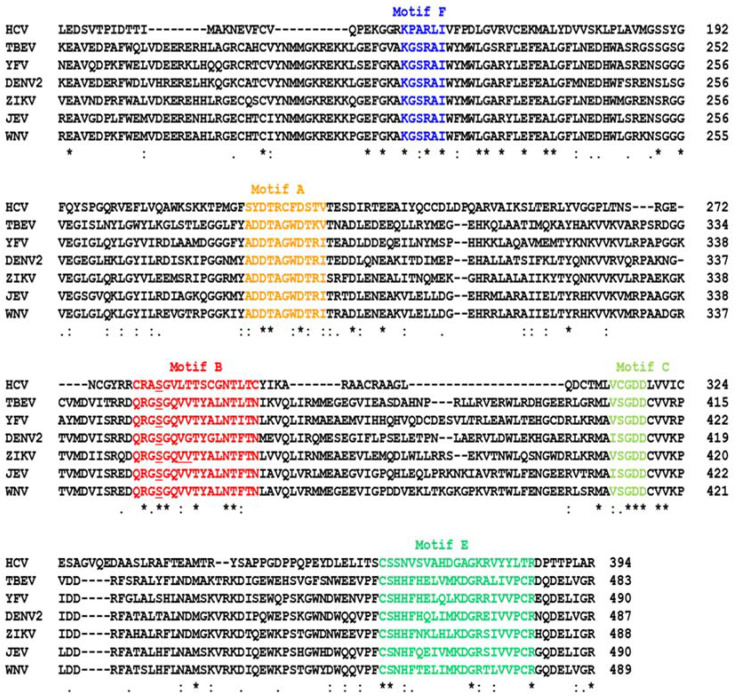
The conserved motifs of the RdRP sequences in representative members of the Flaviviridae family. While HCV is prototypical of the *Hepacivirus* genus of this family, the others belong to the Flavivirus genus in which each virus is the namesake prototype of a cluster of viruses designated as “complex” such as the “Dengue virus complex” and the “yellow fever virus complex”. Note that the flavivirus NS5 sequences are very similar, whereas the lone Hepacivirus in this alignment is divergent, which affected the sequence alignment. The catalytic regions of the *Flaviviridae* NS5 sequences were aligned by Clustal Omega [35]; asterisk (*), colon (:), and period (.) respectively indicate identical, strongly similar, and weakly similar residues. In numbering the residues, the putative N-terminal residue of the processed NS5, liberated from the respective precursor polyprotein, was taken as 1. The motifs are color coded as well as named overhead. Motif G, which is upstream of Motif F, is not shown here, primarily because it is the most diverse in sequence and is characteristic of each virus. The loss of sequence similarity between the HCV and the other viruses can be seen here, going towards the N-terminal side of Motif F. Of note, the catalytic region of NS5, shown here, is harbored in the C-terminal end of the NS5 polypeptide, whereas the N-terminal region encodes the methyltransferase activity that adds the 5’-cap to the nascent product RNA chain. The latter is not shown for lack of relevance, as it is not targeted by the nucleoside analogs reviewed here. The coronaviral RdRP (NSP12) is also not shown here, because its primary structure is substantially dissimilar from the flaviviral ones; however, its 3D architecture and motif arrangement are similar. The underlined Ser in Motif B corresponds to Ser282 in HCV NS5B, which is often found mutated to Thr in NA-resistant HCV isolates (Section 2.2.1). The GenBank accession number of the sequences, presented in the alignment order, are: TBEV AFV48384.1; YFV = NP_041726.1; DENV2 QFS19150.1; ZIKV = QOF88708.1; JEV = ASW22243.1; WNV = ADT91913.1.

At this point, it is relevant to describe the conserved sequence features of viral RdRP, especially the sequence motifs of the active site [30,31]. Specifically, sequence similarity searches and dissection of function have led to the recognition of seven motifs (Figure 1), designated Motif A through Motif G, which are collectively involved in the various aspects of RdRP function such as coordination with the Mg^+2^ ion and interaction with ribonucleotides and polynucleotide chains. The viral RdRPs also display conserved 3D structures that have been architecturally likened to a human right hand with palm, thumb, and finger domains, where the seven motifs, located in specific domains, play their respective roles in RNA replication. A snapshot of these motifs in selected flaviviral RdRPs are shown here, but there are several outstanding reviews that present them in much more detail, which the readers can consult [31,32,33,34].

### 2.1. Remdesivir: Broad-Spectrum Antiviral Role

The various derivatives of remdesivir and their bioconversion are depicted in Figure 1. Historically, the nucleoside form of remdesivir, dubbed GS-441524, was discovered from the screening of nucleoside analogs against multiple emerging RNA viruses [29,36].

**Figure 2:**
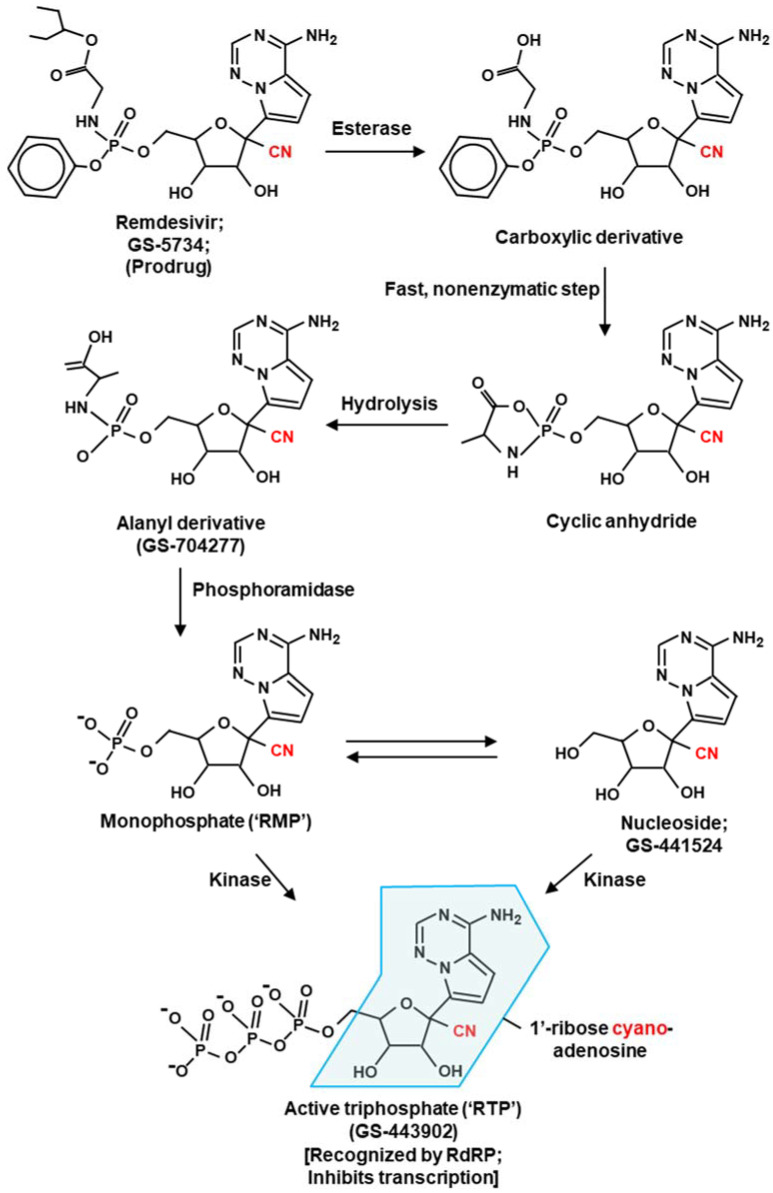
Metabolic activation of remdesivir [5,6,7]. This schematic depicts all the major steps of bioconversion of GS-5734, commonly known as remdesivir, into GS-443902, the final ATP analog that mimics ATP and is recognized by RdRP. In the final structure, the adenosine analog part is boxed in light green. The key cyano substitution (-CN) at the 1’ position of the ribose ring is shown in red; this CN is the primary reason behind the stalling of RdRP as it collides against Ser861 in the RNA channel of the RdRP, thus inhibiting viral transcription.

The efficacy of remdesivir against the Ebola virus (EBOV) in West Africa [8] led to a more extensive investigation that identified GS-5734, the “prodrug” version of GS-441524 (Figure 2), which was also effective against EBOV. GS-5734 was later renamed remdesivir, and various investigators found it to possess broad antiviral activity against many RNA viruses (Table 1), notably, EBOV, Marburg virus, respiratory syncytial virus (RSV), HCV, several paramyxoviruses, and the coronaviruses MERS-CoV and SARS-CoV. When tested specifically against purified SARS-CoV-2 RdRP in vitro, remdesivir showed strong inhibition [8,10,11,12,13]. Collectively, these studies also showed inhibition of several other viral RdRPs by remdesivir.

#### Remdesivir—Molecular Mechanism of Action

Much of our knowledge in this area is owed to the establishment of a partially reconstituted coronavirus (CoV) transcription in vitro. The RdRP holoenzyme of CoV is a large multisubunit complex consisting of 16 nonstructural proteins (Nsps), produced by the proteolytic processing of two large polyproteins early in the virus infection, resulting from the translation of the positive-sense genome RNA by the host translational machinery [37,38,39,40]. The major subunit, Nsp 12, displays RdRP activity in vitro but requires the complex of Nsp7 and Nsp8 for full catalytic activity, the mechanism of which is still unclear [41,42,43,44]. The other Nsps of the holoenzyme provide accessory activities that are required in vivo for optimal and accurate replication. Notably, the helicase activity of Nsp13 unwinds the template–product RNA duplex, and Nsp14 acts as the proofreading 3’-exonuclease, which promptly excises any misincorporated nucleotide in the product RNA caused by the error-prone RdRP [41,45,46,47]. Nsp10 acts a cofactor and stimulates the exonuclease activity of Nsp14. Thus, a minimal coronaviral transcription can be reconstituted in vitro using only three polypeptides, namely, Nsp12, Nsp7, and Nsp8 [10,12,42,44,48,49]; nonetheless, Nsp12 alone is often loosely referred to as RdRP because of its dominant role in transcription and for the sake of brevity.

**Figure 3:**
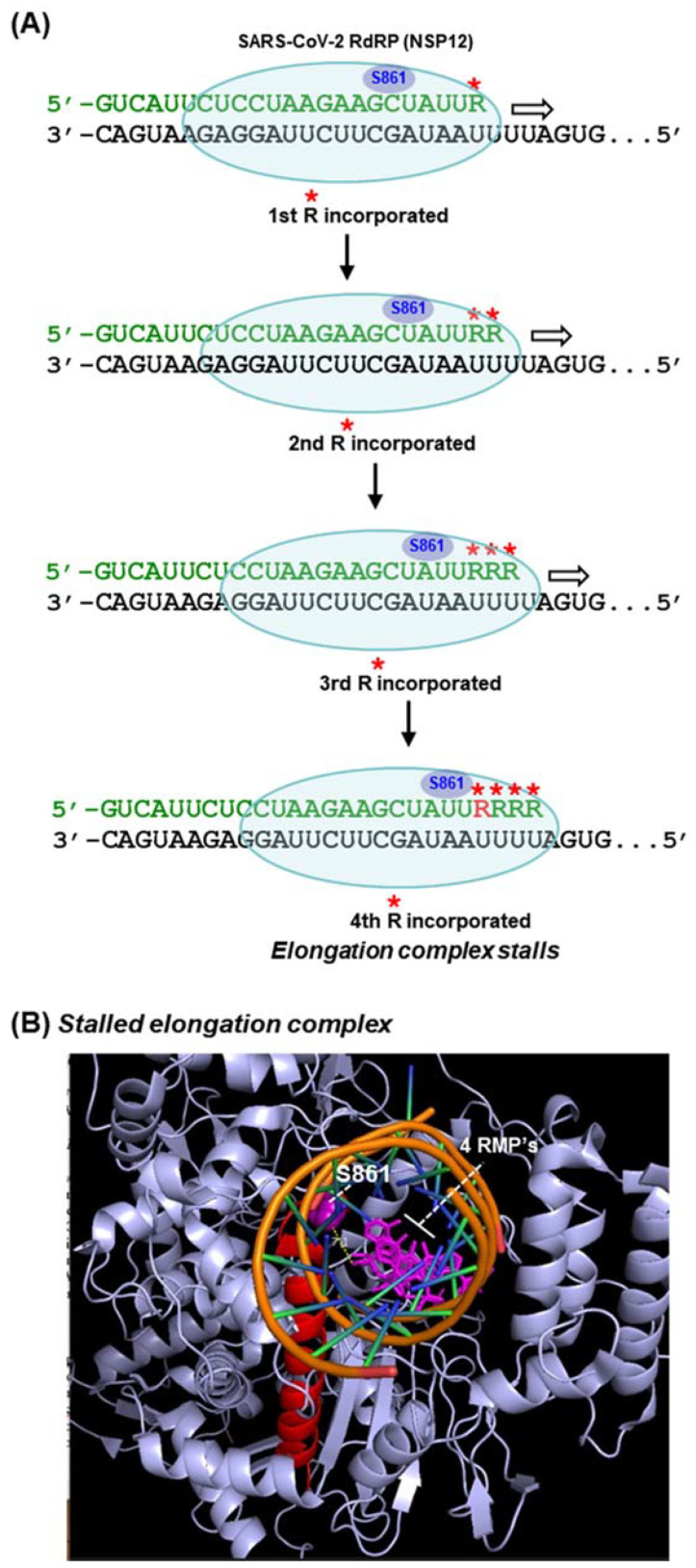
(**A**) Schematic drawing of stepwise transcription elongation in SARS-CoV-2. The RdRP (Nsp12) is shown as an ellipse; the 2’-CN substituents of R are indicated by asterisks (red). The thick arrowhead indicates the direction of RdRP translocation and growth of the product strand. As shown, the elongation complex stalls after four remdesivir monophosphates (R) are incorporated in the product strand (green color), generated by the copying of the template strand (black color). The translocating RdRP (light green oval) stalls when the 4th incorporated remdesivir (RMP) clashes with Ser861 in the RdRP catalytic tunnel [10,12,13,48,52]. Remdesivir—and in some cases, sofosbuvir (Section 2.2)—may also inhibit the RdRP of several other viruses [13] by a similar mechanism (see later). (**B**) Actual 3D structure (7l1f) of the stalled ternary complex, the relevant portion of which is presented in PyMol display. The Nsp12 portions are bluish gray in ribbon display, the RNA strand with H-bonds is depicted as an orange helix and green sticks, the 4 RMP moieties are pink, and the blocking Ser861 is labeled and is located within Motif B, which is mostly helical and in red.

Several studies using this system have suggested that the predominant mechanism of remdesivir action is “delayed” chain termination following incorporation of the remdesivir monophosphate (RMP; Figure 3) in the product RNA chain in place of AMP rather than immediate cessation of transcription [8,9,10,12,48,50].

This is clearly different from the action of 3’-deoxy analogs, such as cordycepin, where the termination is immediate and the next nucleotide cannot be added, as mentioned earlier. In the case of remdesivir, elongation appears to gradually slow down following incorporation; kinetic studies in vitro, using a synthetic RNA template and purified RdRP of SARS-CoV-2, demonstrated stalling of the RdRP translocation after 3–4 additional nucleotides are incorporated following the first RMP moiety [9,10,48]. Determination of the cryo-EM structure of the stalled elongation complex revealed that stalling is due to a block in translocation due to the steric clash between the cyano group of RMP (Figure 2) and the Ser861 side chain of the RdRP [12].

When increasing concentrations of regular nucleotides were added in the reaction, remdesivir-induced termination was gradually abolished, and the RdRP eventually transcribed the full-length product [10,11,51]. Thus, the stalling was a kinetic pause of the elongation complex [52] rather than a total, irreversible termination. In support of the steric clash mechanism, the stalling was also suppressed by mutating Ser861 in the recombinant SARS-CoV-2 Nsp12 to Gly, the amino acid with the smallest side chain (-H). With the substitution with Ala, which has a methyl side chain that is of intermediate size, between the hydroxy of Ser and hydrogen of Gly (H < -CH3 < -OH), only partial stalling was observed [28]. Similar observations were also made for the EV71 RdRP, where the residue equivalent to S861 of the SARS-CoV-2 Nsp12 is S417. In vitro studies revealed that this residue is involved in steric clash with the incorporated RMP, causing delayed stalling of the EV71 RdRP, also three nucleotides downstream. The remdesivir-induced stalling did not occur in the S417G mutant EV71 RdRP [53].

Interestingly, these studies unraveled a second mechanism in which remdesivir was capable of causing misincorporation [51]. In these studies, as the steric clash was eliminated, the S861G mutant RdRP continued RNA synthesis even after remdesivir moieties were incorporated. In the resultant RNA product, R substituted A in several places, the extent of substitution depending on the RTP: ATP ratio in the reaction. Subsequently, when the RMP-incorporated RNA was the template, the efficiency of incorporation of the complementary UMP opposite the templated RMP was compromised, providing another opportunity to inhibit viral RNA synthesis. Structure modeling and mutational analysis suggested that the UMP in the nascent RNA, complementary to the template RMP, is improperly positioned because of a steric clash with Ala558. Taken together, remdesivir inhibits SARS-CoV RdRP by two mechanisms: (a) when incorporated in the nascent RNA it promotes stalling within ~3 nucleotides due to the steric clash with Ser861; (b) if the stalling is suppressed and the RMP-containing product RNA is subsequently used as template, the UMP in the nascent RNA now clashes with Ala558 of RdRP, again leading to inhibition. Fundamentally, both mechanisms involve a steric clash between a nucleotide in the viral RNA and a specific amino acid residue of the viral RdRP, caused directly and indirectly by remdesivir incorporation.

The majority of the SARS-CoV transcription reactions that showed the inhibitory activity of NAs in vitro employed the minimal heterotrimeric complex, composed of Nsp12, and the accessory factors Nsp7 and Nsp8, without the Nsp14-Nsp10 3’-exonuclease. Unfortunately, when the exonucleases were present, they proofread and removed incorporated NAs, including the delayed terminator, remdesivir, and the immediate terminator, sofosbuvir, although the latter was somewhat more resistant to exonuclease, probably due to the fact of its structural distinction from remdesivir [54]. The relative success of remdesivir in inhibiting SARS-CoV in cell culture may be due to the delayed termination that moves the RMP to internal positions, thereby making it exonuclease resistant by the time it exits the RdRP catalytic pocket. As previously conjectured [12], the outcome is largely determined by the kinetic competition between elongation and the ability of the exonuclease in the RdRP holoenzyme to contact the 3’-terminal RMP immediately after incorporation. The difference in Km between remdesivir and ATP and the ratio of their concentration will certainly be the other determining factors. Nonetheless, the inclusion of an exonuclease inhibitors, such as pibrentasvir, protected the 3’-NAs from excision, and the transcription termination was improved [55]. Viral cell culture studies also demonstrated the significant synergy of the drug combination, suggesting that this could be a more effective therapeutic regimen in COVID-19 treatment than the NA drug alone.

As stated earlier, the mechanism of the transcription inhibition by remdesivir was also investigated in several other RNA viruses, whereby most of them appeared to follow the SARS-CoV-2 paradigm of delayed termination. In a recent study, remdesivir and 4’-fluorouridine (4’-FlU, a U analog) both inhibited in vitro transcriptions, reconstituted with the minimal RdRP of RSV and SARS-CoV-2 [13]. While the SARS-CoV-2 RdRP was composed of Nsp12-Nsp7-Nsp8, as mentioned before, the RSV RdRP comprised the large subunit L and its transcription factor, phosphoprotein P [56,57,58]. In both cases, the delayed stalling of transcription occurred ~3 nucleotides after the 4’-FlU incorporation; in parallel reactions, the well-characterized remdesivir was used against SARS-CoV-2 RdRP as a positive control for the delayed stalling [13]. The therapeutic efficacy of 4’-FlU against RSV was confirmed in the infection of human airway epithelial cells in air–liquid interface culture as well as in the Balb/cJ mouse model. However, the mechanism of the delayed stalling by 4’-FlU remains unknown. However, the exact amino acid residue involved the blocking of RdRP in these viruses remains unmapped and would ideally need structural studies of stalled complexes as was conducted in SARS-CoV-2.

### 2.2. Sofosbuvir

**Figure 4:**
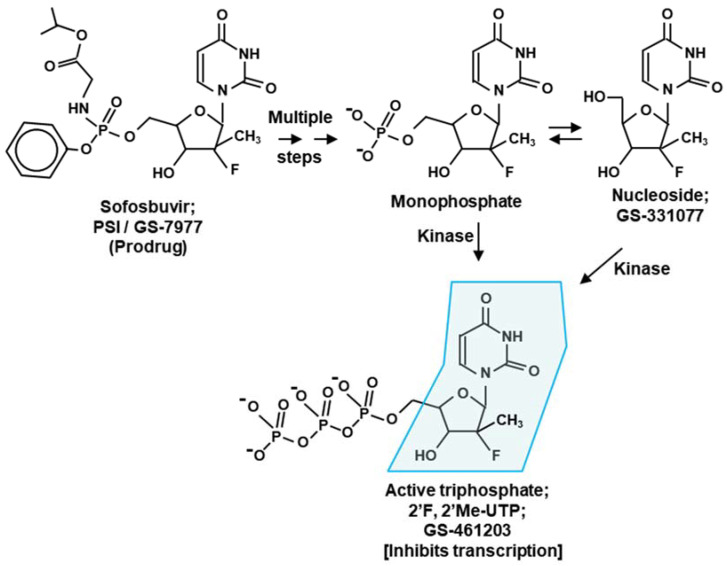
Metabolic activation of sofosbuvir (GS-331077) through the intermediate nucleoside form. The steps are shown only briefly, as they are very similar to those for remdesivir, shown earlier (Figure 2). The final triphosphate form mimics UTP and is recognized by RdRP to be incorporated in the product RNA in place of U, which results in transcription termination [62,63]. The corresponding CTP analog (triphosphate from PSI-6130), discussed below (Section 2.2.1), is also generated essentially by the same pathway [62].

Like remdesivir, sofosbuvir (GS-331077), a uridine analog, terminates transcription when incorporated into the viral RNA. Initially licensed for use in HCV infection (Table 1), sofosbuvir was evaluated as a broad-spectrum inhibitor against several other flaviviruses, which showed significant antiviral activity (hence, the term “direct-acting antiviral”) [24,26,59,60,61,62]. Collective evidence reveals viral RdRP, namely, flaviviral NS5, as the target of inhibition. Sofosbuvir is a prodrug that is metabolized to the active antiviral agent GS-461203 (2’-deoxy-2’-α-fluoro-β-C-methyluridine-5’-triphosphate or 2’F-2’C-Me-UTP in short) [5,63] (Figure 4). GS-461203 serves as a defective substrate for the RdRP, which is the viral RNA polymerase, thus acting as a chain terminator in viral RNA synthesis [63].

#### 2.2.1. Sofosbuvir: Molecular Mechanism of Action

Unlike the delayed stalling effect of remdesivir, sofosbuvir stops the elongating RdRP immediately without allowing the next nucleotide to be added. In the absence of a 3D structure of the sofosbuvir–RdRP complex, the molecular mechanism of how exactly sofosbuvir promotes termination remains unclear. Nonetheless, a similar nucleotide analog, 2’-deoxy-2’-α-fluoro-β-C-methylcytidine, or 2’F-2’C-Me-CTP for short (the prodrug is R7128; nucleoside is PSI-6130), acts as a transcription terminator and inhibits flaviviruses likely because its 2’ methyl group (on ribose) causes a steric clash with an incoming NTP [62], which led to the suggestion that sofosbuvir may follow a similar mechanism. As mentioned earlier, the conserved Ser in Motif B (Figure 1) is located in a similar position as Ser861 on SARS-CoV-2 RdRP in the 3D structure (Figure 3).

Several other amino acid residues in the RdRP active site are also relevant in the NA mechanism. Of these, Ser282 of HCV is the best studied and deserves mention here, especially in regard to the antiviral activity of the C-analog, 2’-C-Me-CTP, which showed proof-of-concept clinical efficacy in HCV-infected patients [64,65]. Nonetheless, the clinical studies also generated drug-resistant mutations in HCV, a class of which mapped to Ser282 of the HCV NS5 (i.e., RdRP), whereby the Ser mutated to a Thr (S282T) [66,67,68,69]. Several subsequent studies revealed a mechanism for the resistance of the S282T HCV, which also shed light on the structure and function of the RdRP active site. Briefly, RdRP assays with defined templates showed that 2’-C-Me-CTP, indeed, directly inhibits RNA synthesis, and that the S282T is considerably resistant [70]. Structural studies revealed that this is due to the side chain of Thr (i.e., -CH(CH3)OH) with its methyl group, is larger than that of Ser (-CH_2_OH). In the architecture of the active site, S282 is positioned directly below the terminal nucleotide of the primer strand, such that a Thr in this position blocks the incoming 2’-C-Me-CTP more than the normal CTP [70]. 2’-O-methyl-CTP, also an inhibitor of HCV RdRP (NS5B), to which the S282T mutant is also resistant due to the steric clash, but the structural mechanism is somewhat different in the details, as suggested by model building in silico [71]. In this mechanism, the 2’-O-methyl substitution changes the natural 3’-endo conformation to 2’-endo, colliding it not only with the backbone of Ser282 but also with the methyl group of Thr287 [71].

The prime location of Ser282 has drawn the attention of antiviral researchers. Moreover, it is located in Motif B, which is highly conserved in all RdRPs, particularly in flaviruses, as shown in the multiple sequence alignment (Figure 1). Based on the above, it could be conjectured that mutation of this Ser to Thr in flaviviruses may also lead to flavivirus resistance to NAs, such as sofosbuvir, that also have bulky 2’ substitutions. Indeed, in a reverse genetic study in which the analogous Ser of ZIKV polymerase (S352 in our numbering; Figure 1) was mutated to Thr and the inhibition of the recombinantly expressed mutant by sofosbuvir was tested in a cell-free biochemical assay, the mutant polymerase exhibited sofosbuvir-resistance [66]. However, a subsequent study has pointed to an important role of other residues of Zika RdRP in actual selection of drug-resistance [24]. In this study, ZIKV-infected human hepatoma cells were grown in culture in the presence of a range of sofosbuvir concentration and the NS5 (RdRP) gene of the resistant isolates were sequenced. Interestingly, none of the isolates from three independent experiments contained the aforementioned mutation in Ser; however, several other sites were found mutated, including a nearby Val within Motif B, which is conserved in flaviviruses (Val355; Figure 1), but not in HCV, emerged [24]. The authors correctly realized the importance of these data for anti-ZIKV drug development.

**Figure 5:**
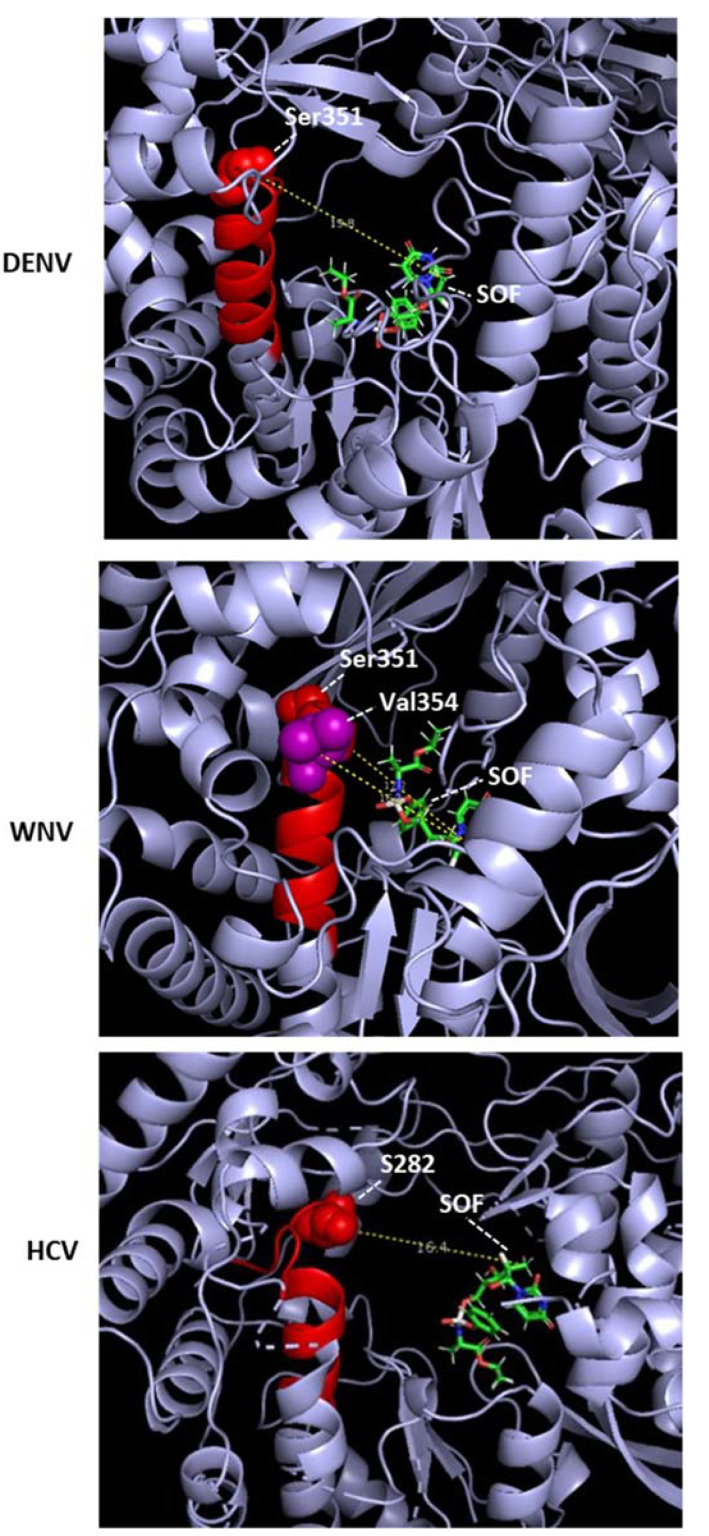
Structural location of sofosbuvir, bound inside viral RdRP catalytic pockets, generated from published structures using the EDock program [72] as described in Section 2.2.1. The structures are displayed by PyMOL. The RdRP is represented by the bluish gray ribbon display; the relevant Ser and Val are labeled and are located within Motif B, which is mostly helical and red in color; the single molecule of sofosbuvir (SOF) is shown as sticks. The “Measurement” function of PyMol was used to determine the theoretical atomic distance between the indicated amino acids in Motif B and the 2’C-methyl group of the drug (Figure 4), since this methyl group is the sole difference between the drug and the normal UTP. To clearly view the drug and the distance markers, each structure was rotated as needed; in addition, the “Hide” function of PyMol was used to remove portions of the catalytic channel, consisting mostly of loops.

Since the 3D structures of sofosbuvir–RdRP complexes have not been solved for other flaviviruses, I conducted a modeling of sofosbuvir in the catalytic tunnel of DENV and WNV NS5 in silico using EDock [72]. The top poses of the model revealed atomic distances between sofosbuvir and NS5 residues, which were slightly variable due to the multiple possible energy values and drug conformations but revealed a pattern. The Ser residues that are a HCV-S282 analog in multiple sequence alignment of the flaviviruses, as presented earlier (Figure 1), are DENV2, S351; WNV, S351. In EDock simulations, the following 3D structures of Apo NS5 were used as PDB files: DENV2, 5ZQK; WNV, 2HFZ. The sofosbuvir structure was from PubChem (pubchem.ncbi.nlm.nih.gov (accessed on 24 September 2022); Compound CID 45375808). In general, S351 was far from sofosbuvir, making it unlikely that it would clash with sofosbuvir. For example, the distances between S351 and sofosbuvir were DENV2, 19.8 and WNV, 17.2 (Figure 5). Another WNV residue in Motif B, namely, V354, is 16.2 A away from sofosbuvir, also ruling out atomic contact (Figure 5). In a similar EDock application on HCV NS5B and sofosbuvir, the S282–drug distance was also large, averaging 16.4 A in different simulations. It should be noted that the published 3D structure showing S282–drug proximity (3.9A) and the possibility of a clash is represented by PDB 4WTG [73], and it is actually that of a ternary complex that also contained RNA bound in the vicinity, which could affect the structure and, hence, the binary distance. To our knowledge, the 3D structure of the sofosbuvir–RdRP complex has not been solved for any other RNA virus. Nonetheless, the sofosbuvir-resistant ZIKV mutant described above [24] was V354I, in Motif B (Figure 1).

It is, therefore, important to keep in mind that if sofosbuvir indeed inhibits flaviviral RdRP by blocking the elongating RdRP, the location of the block may not exactly coincide with that of 2’-C-Me-CTP in HCV NS5B, but other amino acids in the vicinity may be involved. As pointed out previously, the amino acid residues K51, S282, T286, and M289 are also close to the active site of the HCV polymerase and, hence, could be involved in the presumptive steric block. Perhaps the most relevant localization of the block would be viewed in the 3D structure of an actual drug–stalled elongation complex, as was conducted for remdesivir [12].

Clearly, if sofosbuvir-resistant mutation(s) appear in DENV and other flaviviruses in the future, it will be worthwhile to map its location in the primary structure and, at the same time, determine the 3D structure of cocrystals, obtained by cryo-EM or crystallography to complement the studies.

#### 2.2.2. Sofosbuvir: Potential Side Effect

Remdesivir and sofosbuvir hold the distinction of having received fast-track “emergency authorization” for clinical use in COVID-19- and HCV-infected patients, respectively. A query of their off-target effects led to the search for host genes that are differentially expressed in treated versus untreated individuals. The premise that the drugs may inhibit mitochondrial function received special attention in view of the distant similarity of the active sites of viral RdRP and mitochondrial RNA polymerase (mtRP). However, an assay of the diverse mitochondria function revealed little inhibition by remdesivir at clinically relevant concentrations [74,75]. In transcription assays in vitro [76], mtRP did not incorporate 2’-C-methyl-2’-fluoro-UTP, the active triphosphate metabolite of sofosbuvir (Figure 4). Thus, these analogs have negligible off-target effects, validating their emergency authorization. 

Although the evolutionary origin of RdRP genes remains unknown, it occurs primarily in nonretroviral RNA viruses, as presented here. In eukaryotic cells, the RdRP activity is responsible for the amplification step of RNA interference (RNAi), found in plants and fungi [77,78]. RdRP homologs could not be detected in most animals, including human and fruit fly, even though RNAi occurs in them. An early study, for example, showed that cordycepin (3’-dA), which inhibits viral RdRP, such as that of Dengue virus (Section 1 and Table 1), did not inhibit RNAi in mammalian oocytes [77]. In summary, viral RdRP remains a viable target for antiviral drugs.

### 2.3. Molnupiravir

**Figure 6:**
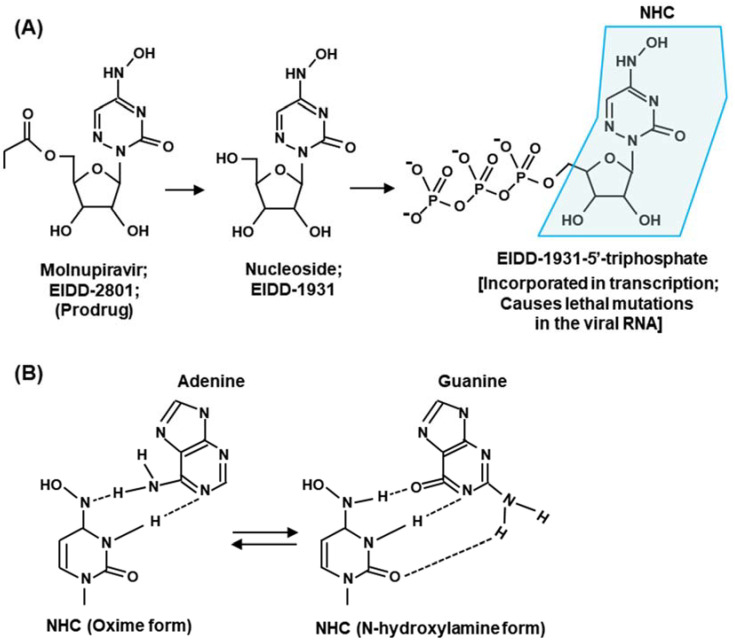
(**A**) Metabolic activation of molnupiravir (EIDD-2801; or MK-4482) through the intermediate EIDD-1931. The final triphosphate form mimics CTP and is recognized by RdRP to be incorporated in the product RNA in place of C. The NHC (β-D-N4-hydroxycytidine) moiety is boxed in light green [79]. (**B**) The tautomeric forms (i.e., oxime and N-hydroxylamine) of NHC are shown hydrogen bonded with purine bases A and G, respectively.

Molnupiravir (EIDD-2801) [14,79] is representative of NAs that promote widespread mutations in viral RNA. Molnupiravir is metabolically converted into a cytidine nucleoside (C) analog, β-D-N4-hydroxycytidine 5’-triphosphate (NHC-TP; also known as EIDD-1931) (Figure 6A).

Unlike remdesivir, the NHC-monophosphate, when incorporated in the product RNA, does not terminate transcription [16,63,80] but promotes mutations when used as a template in the subsequent cycle of RNA synthesis. In this mechanism, NHC also exemplifies nucleoside analogs that tautomerize between two alternative structures in equilibrium, in this case cytosine and uracil (Figure 6B). Depending on the structure it attains, it can then form Watson–Crick hydrogen bond with either G or A. The hybridization with G results in wild-type pairing, but an A in the template would pair with U in the next cycle to cause a GC to AU mutation. As a result, NHC causes mutation approximately half of the time it is incorporated, which forms the basis of lethal mutagenesis and resultant eradication of the virus. Likewise, Flavipiravir can mimic both A and G and, therefore, can base pair with U or C [81].

## 3. Summary Rules and Future Directions

### 3.1. Summary Rules for Antiviral Nucleoside Design

Based on what we know so far, in order for a nucleoside analog (NA) to target viral RdRP and act as an antiviral, it needs to meet two fundamental criteria.

(i) Acceptance by RdRP: The NA should be able to enter the NTP-binding domain of the RdRP where the normal nucleotide binds for transcription. Obviously, NAs with bulky substitutions or very different chemical properties will be unacceptable. This is why the prodrug does not work. First, it has a large substituent, introduced for improved pharmacology [82]; second, it lacks the 5’-triphosphate group of the ribose. The substituent is, therefore, removed and the nucleoside is tri-phosphorylated for recognition of the analog by the RdRP;

(ii) Incorporation in product RNA: The NA needs to be incorporated in the growing RNA chain, following which one of several things may happen: (a) The incorporated NA prevents joining with the next NTP due to the lack of 3’-OH (as in 3’-dA, i.e., cordycepin). For the same reason, 3’-OH cannot be derivatized, since it is necessary for the 3’-5’ phosphodiester bond. (b) Alternatively, the NA with a normal, extendable 3’-OH can block transcription for several other reasons, such as a spatial clash with an amino acid side chain in the catalytic tunnel of the RdRP, as in remdesivir and sofosbuvir. (c) Lastly, it allows for transcript elongation, but its base-pairing property is different from that of the original nucleotide that it replaced. Subsequently, this leads to mutant viral RNA, with a lethal effect on the virus.

(iii) Generalized ProTide technology: The prodrug forms of NA inhibitors, as described here, and other similar derivatives are often referred to as ProTide (Prodrug-Nuclotide) technology [82,83,84,85]. The goal here is to block the phosphate group of the nucleotide so that the resultant prodrug is neutral at physiological pH, which improves cellular uptake. As shown for remdesivir and sofosbuvir [86,87], the prodrug modification often consists of aryloxy substitution, which also increases lipophilicity and further enhances membrane uptake. Thus, the most commonly used ProTide modification cages the anionic phosphate through an N-linked amino ester and an O-linked aromatic phosphoester using phosphoramidate chemistry, such that release of the active drug requires consecutive action of an esterase followed by a phosphoramidase (Figure 2 and Figure 4). However, many variations of this theme have been explored [85,86], and it is recommended that such substitutions are screened for the optimal stability, uptake, and overall pharmacokinetics of a candidate nucleoside/nucleotide analog.

Lastly, regardless of all our efforts, drug-resistant viral mutations have appeared throughout history including many against NAs; some of them have been described before, such as the S282T mutant of HCV and the sofosbuvir-resistant ZIKV mutant. Currently, this is circumvented by multidrug combination therapy and screening with novel analogs. It is fair to assume that this evolutionary battle between virus and man will continue. It is hoped that this critical review, which includes structural discussions, will inspire the design and evaluation of additional nucleoside analogs as antiviral candidates for use in the clinic as well as in the research laboratory.

### 3.2. Relevant Tissue Culture System: A Suggestion for Future Research

Mammalian cells are traditionally cultured in two-dimensional monolayers for initial studies of virus infection and drug sensitivity screening. However, it has been increasingly acknowledged that a three-dimensional cell culture may better approximate metazoan tissues and organs and may be suitable for diverse studies such as “epithelial–mesenchymal transition”, drug uptake, and virus growth [88,89,90,91,92]. Many viruses show stringent preference for either the apical or the basolateral membrane of the cell [93,94], which are better studied in 3D cultures. We and others have noted dramatic differences in the growth pattern and the cytoskeletal structure of the epithelial cell lines A549, MDCK (Madin–Darby canine kidney), and Caco-2 in polarized cultures grown on Millipore membranes versus these same cells in standard monolayers [95] (see Supplementary Materials Figure S1). The tight cell–cell adhesions, as seen here, simulating a multicellular tissue, is often referred as “branching morphogenesis”, particularly well-studied in MDCK cells [96]. Several alternative growth systems have also been suggested, and I recommend that they be adopted in the studies of the nucleoside analogs as well, since animal models are significantly more difficult, labor-intensive, expensive, and of questionable ethics [97].

## Data Availability

Not applicable.

## Supplementary Materials

The supporting information can be downloaded at: https://www.mdpi.com/article/10.3390/ijms232012649/s1.
