# Supplementary material for: Inhibition of Viral RNA-Dependent RNA Polymerases by Nucleoside Inhibitors: An Illustration of the Unity and Diversity of Mechanisms
Source: Int J Mol Sci. 2022 Oct 21;23(20):12649. doi: 10.3390/ijms232012649 (PMC9604226; doi:10.3390/ijms232012649)
Supplement: Supplementary file 1 [file ijms-23-12649-s001.zip › ijms-1962821-supplementary.pdf]

Supplementary Material

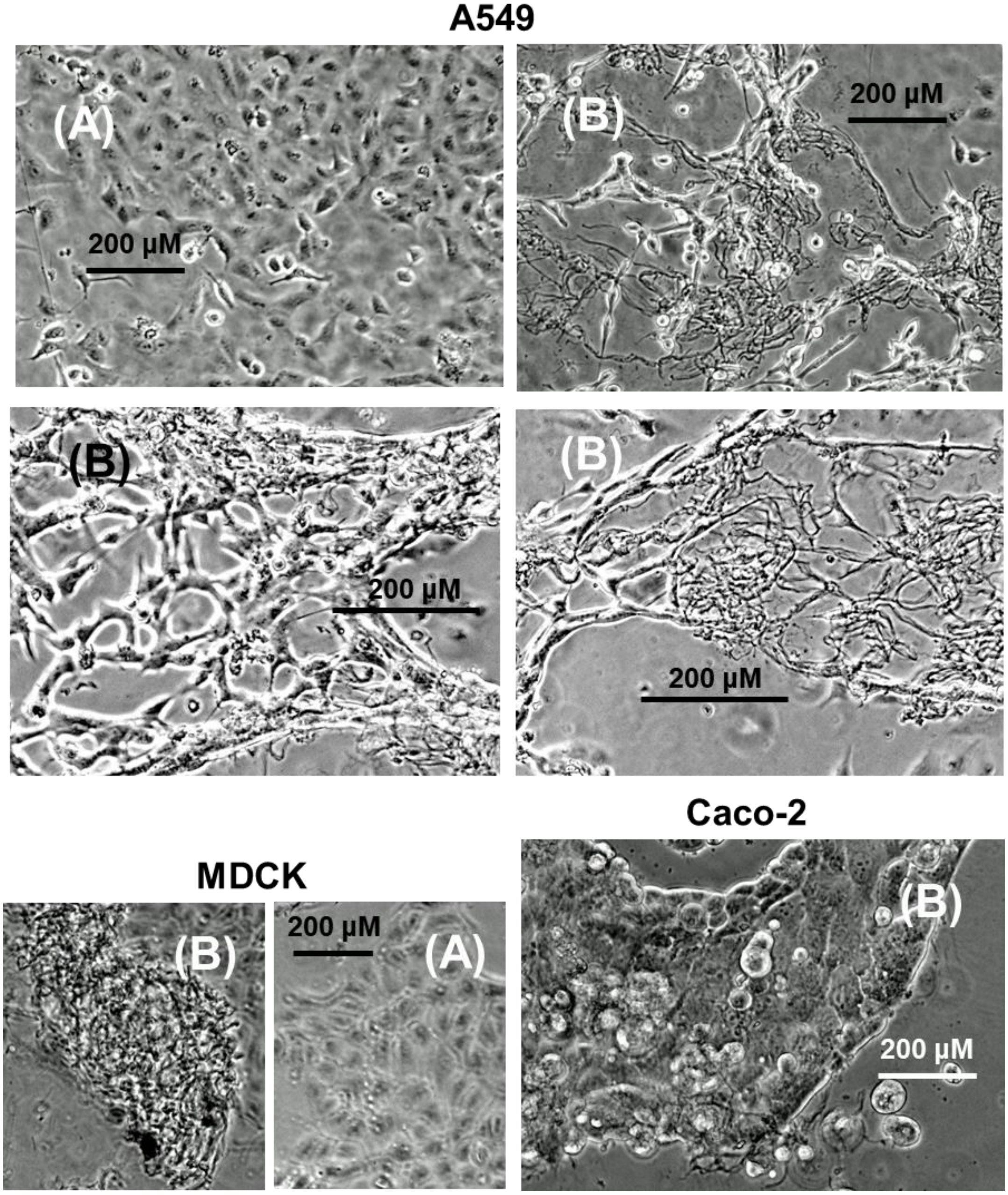

Figure S1: Growth of common cell lines on standard plastic plates versus membranes. This supplementary Figure is companion to Section 3.2. in the main paper and shares the same reference numbers. The indicated cells (A549, MDCK, Caco-2) were grown either on **(A)** regular plastic 6-well plates or on **(B)** semipermeable Matrigel membranes (Millipore Sigma) [89], for comparison. Phase contrast images were captured as before [95]. The length scale bar is shown for each image. Some images are shown at two magnifications for better viewing of cell morphology as well as growth pattern. Note the complex growth pattern of all cell types in Matrigel-grown cultures.
